# Rituximab Monotherapy Is Effective as First-Line Treatment for Granulomatous Lymphocytic Interstitial Lung Disease (GLILD) in CVID Patients

**DOI:** 10.1007/s10875-023-01587-4

**Published:** 2023-09-27

**Authors:** Giulio Tessarin, Manuela Baronio, Luisa Gazzurelli, Stefano Rossi, Marco Chiarini, Daniele Moratto, Silvia Clara Giliani, Maria Pia Bondioni, Raffaele Badolato, Vassilios Lougaris

**Affiliations:** 1 Department of Clinical and Experimental Sciences, Pediatrics Clinic and Institute for Molecular Medicine A. Nocivelli, University of Brescia and ASST Spedali Civili of Brescia, Piazzale Spedali Civili 1, 25123 Brescia, Italy; 2 Department of Molecular and Translational Medicine, Institute for Molecular Medicine A. Nocivelli, University of Brescia, Brescia, Italy; 3 Diagnostic Department, Flow Cytometry Laboratory, ASST Spedali Civili of Brescia, Brescia, Italy; 4 Department of Medical and Surgical Specialties, Pediatric Radiology, University of Brescia and ASST Spedali Civili of Brescia, Brescia, Italy

**Keywords:** Common variable immunodeficiency, inborn error of immunity, granulomatous lymphocytic interstitial lung disease, rituximab, anti-CD20 monoclonal antibody, lung, CVID, GLILD, T lymphocyte, B lymphocyte

## Abstract

Granulomatous lymphocytic interstitial lung disease (GLILD) represents a fatal immune dysregulatory complication in common variable immunodeficiency (CVID). Evidence-based diagnostic guidelines are lacking, and GLILD treatment consists in immunosuppressive drugs; nonetheless, therapeutical strategies are heterogeneous and essentially based on experts’ opinions and data from small case series or case reports.

We aimed to evaluate the efficacy and safety of first-line Rituximab monotherapy for CVID-related GLILD, by assessing symptoms and quality of life alterations, immunological parameters, pulmonary function tests, and lung computed tomography.

All six GLILD patients received Rituximab infusions as a first-line treatment. Rituximab was administered at 375 mg/m^2^ monthly for six infusions followed by maintenance every 3 months; none of the patients experienced severe adverse events. Symptom burden and quality of life significantly improved in treated patients compared to a control group of CVID patients without GLILD. Rituximab treatment indirectly caused a trend toward reduced T-cell activation and exhaustion markers sCD25 and sTIM-3. Lung function improved in treated patients, with statistically significant increases in TLC and DL_CO_. Lung CT scan findings expressed by means of Baumann scoring system displayed a reduction in the entire cohort.

In conclusion, first-line monotherapy with Rituximab displayed high efficacy in disease remission in all treated patients, with improvement of symptoms and amelioration of quality of life, as well as restoration of PFTs and lung CT scan findings.

**Supplementary Information:**

The online version contains supplementary material available at 10.1007/s10875-023-01587-4.

## Introduction

Common variable immunodeficiency (CVID)-affected patients suffer from increased susceptibility to infectious episodes; in addition, up to 70% of CVID patients may also develop non-infectious complications, such as autoimmunity, lymphoproliferation, granulomatous disease, and increased risk in developing malignancies [1, 2]. Granulomatous lymphocytic interstitial lung disease (GLILD) represents a non-infectious immune dysregulatory complication which occurs in 8–20% of CVID patients, with a massive impact on patients’ quality of life (QoL) and life expectancy [3, 4]. GLILD has been recently defined in a consensus statement by the British Lung Foundation/United Kingdom Primary Immunodeficiency (UK-PID) Network as a “distinct clinico-radio-pathological interstitial lung disease occurring in patients with CVID, associated with a lymphocytic infiltrate and/or granuloma in the lung” [4]. Evidence-based guidelines are lacking, and current recommendations are mainly based on the UK-PID Network consensus statement: suggested investigations comprise pulmonary function tests (PFTs), chest computed tomography (CT) scan, bronchoscopy, and lung biopsy [4]. Besides optimal immunoglobulin replacement treatment (IgRT) and supportive therapies, the treatment of GLILD consists in immunosuppressive drugs; nonetheless, strong evidences are missing, and GLILD therapeutical strategies are essentially based on less robust data such as experts’ opinions or small case series or case reports [5]. In line with the emerging evidences on the role of B-cell hyperplasia in driving and maintaining GLILD in CVID patients, B-cell depletion via anti-CD20 monoclonal antibody showed good results in the vast majority of the treated patients achieving both disease remission and disease-free maintenance, even though GLILD relapses may occur in those patients with B-cell reconstitution [5, 6]. Nevertheless, all these data are mainly derived from single case reports, and few studies present large cohorts of patient with comprehensive evaluation before and after the provided treatment (such as the large cohort of patients treated with Rituximab and antimetabolites reported by Verbsky and colleagues [7]), thus reducing the quality of the studies and the strength of their evidence.

## Methods

### Study Setting—Population

We conducted a monocentric retrospective analysis on a cohort of pediatric and adult CVID patients to investigate the prevalence of GLILD, the type of treatment provided, and the outcome after the treatment. Patients that have not performed at least one lung CT scan at the time of CVID diagnosis and during the follow-up were not included in this study. All patients have been evaluated at the Immunology Unit of the Pediatrics Clinic of Brescia Children Hospital, ASST Spedali Civili of Brescia (Brescia, Italy), and University of Brescia between 1 January 1990 (start of the present study) and 30 October 2022 (end of the present study). A diagnosis of CVID was established according to the ESID Registry working definitions for clinical diagnosis of IEIs [1]. A diagnosis of GLILD was achieved after multidisciplinary evaluation of clinical and radiological data by experienced physicians, PFTs, and bioptic specimens was used when available to corroborate the diagnosis [4]. A pool of 30 age- and sex-matched CVID patients without GLILD diagnosis were used as control group for statistical comparisons.

### Ethics Approval

This study was performed in line with the principles of the Declaration of Helsinki. Written informed consent was obtained from all patients (or their caregivers in case of pediatric patients) included in this study. The study has been approved by the ASST Spedali Civili of Brescia Ethical Committee (NP2972).

### Rituximab Monotherapy

GLILD-affected patients received anti-CD20 monoclonal antibody (Rituximab) intravenous infusions (375 mg/m^2^) monthly for 6 infusions, followed by an every 3-month maintenance scheme; no other immunosuppressive/immunomodulator agents were implemented. Laboratory analysis (complete blood count, serum IgA and IgM levels, lymphocyte immunophenotyping, T-cell activation markers), QoL, PFTs, and lung CT scans were performed before treatment and after 6-month Rituximab infusion to evaluate treatment response (Fig. S1 in this Article’s Online Resource).

### Demographical and Clinical Data—QoL Evaluation

Demographical and clinical data were obtained from retrospective analysis of patients’ medical records. Symptomatic burden and QoL have been measured by the St. George’s Respiratory Questionnaire (SGRQ), a pulmonary disease-specific questionnaire measuring self-reported symptoms and their relationship to activities of daily living and psychological functioning [8]. The SGRQ has 50 items with 76 weighted responses, covering three different domains: symptoms, activity, and impacts (psychosocial) as well as a total score. A minimum change in score of 4 units was established as clinically relevant after patient and clinician testing [9]. The SGRQ was administered at GLILD diagnosis and after 6 months of Rituximab treatment.

### Laboratory Data

Complete blood count and serum IgA and IgM levels were performed at the Clinical Chemistry Laboratory, Diagnostic Department, ASST Spedali Civili of Brescia (Brescia, Italy), using standard techniques. IgG serum levels were not collected as a significant number of patients were already undergoing immunoglobulin replacement therapy (IgRT). Lymphocyte immunophenotyping was performed for all patients at the Flow Cytometry Unit, Diagnostic Department, ASST Spedali Civili of Brescia (Brescia, Italy). All analyses were performed from fresh blood samples drawn in the previous 24 h. Appropriate mixtures of monoclonal antibodies were used to design multicolor panels and stain blood cells according to manufacturer’s protocols. Multicolor tubes were acquired on a FACS Canto II (BD Biosciences) flow cytometer. Data were analyzed with FACS Diva (BD Biosciences) software. A standard T-B-NK panel was used to analyze the main lymphocyte populations. CD4^+^ or CD8^+^ T-cell subpopulation was further characterized as naïve (CCR7^+^CD45RA^+^), recent thymic emigrants (RTE) (CCR7^+^CD45RA^+^CD31^+^), central memory (CCR7^+^CD45RA^-^), effector memory (CCR7^-^CD45RA^-^), and terminal effector memory (CCR7^-^CD45RA^+^). B-cell subsets were differentiated between recent bone marrow emigrants (CD38^++^CD10^+^), naïve (CD27^-^IgD^+^IgM^+^CD21^hi^), switched memory (CD27^+^IgD^-^IgM^-^CD21^hi^), IgM-memory (CD27^+^IgD^+^IgM^+^CD21^hi^), terminally differentiated (CD38^++^CD27^+^CD20^-^), and CD21^low^ cells (CD19^hi^CD21^lo^). Data on main populations (CD3^+^, CD4^+^, CD8^+^, CD19^+^, CD16^+^) were collected and analyzed as absolute numbers (cells/μL); T- and B-cell subpopulations were collected and analyzed as percentages of CD4^+^, CD8^+^, and CD19^+^ cells, respectively. All patients underwent genetic analysis for 320 IEI-related genes via targeted next-generation sequencing (NGS) techniques using Ion Torrent Personal Genome Machine System (Life Technologies Ltd., Paisley, UK). Potential pathogenic variants detected using NGS approach were validated by performing Sanger sequencing.

### Serum Collection and T-Cell Activation and Exhaustion Markers Analysis

The serum of GLILD-affected CVID patients, non-GLILD-affected CVID patients, and healthy donors (HDs) was sampled, collected in sterile tubes, centrifuged at 3000 rpm for 15 min, and stored at−20 °C. At the time of sampling, none of the patients nor the controls had ongoing acute infection, immune modulating medical treatment, and concomitant administration of any vaccination. Serum levels of soluble IL-2Rα chain/CD25 (sCD25) and soluble T-cell Ig and mucin domain-containing protein 3 (sTIM-3) were measured by enzyme immunoassays using commercially available kits (R&D Systems, Minneapolis, MN) according to manufacturer instructions.

### Pulmonary Function Tests

PFTs had been conducted on a Vmax Pulmonary Function Unit (ViaSys, Santa Ana, CA, USA) in accordance with European Respiratory Society (ERS)/American Thoracic Society guidelines [10]. Pulmonary function variables included forced vital capacity (FVC), forced expiratory volume in 1 s (FEV_1_), and total lung capacity (TLC). Diffusion capacity of the lung for carbon monoxide (DL_CO_), adjusted for hemoglobin and alveolar volume (AV), had been measured with the single breath carbon monoxide test; Krogh index (K_CO_) was calculated as DL_CO_/AV. Data are presented as percentage of predicted values according to the ERS 1993 update [11]. PFTs were performed at GLILD diagnosis and after 6 months of Rituximab treatment.

### Chest Computed Tomography and Baumann Scoring System

Digital CT scan DICOM files were collected using the picture archiving and communication system (PACS) software. Lung CT scans were performed at GLILD diagnosis and after 6 months of Rituximab treatment. Lung CT scans were scored according to the Baumann scoring method by one blinded consultant radiologist [12, 13].

### Statistical Analysis

Data were analyzed using the software GraphPad Prism 8. We reported percentage, mean, median, and interquartile range (IQR) as descriptive statistics. Fisher’s exact tests were used for categorical variables. Parametric (Student’s *t*-test) or non-parametric tests (Wilcoxon matched pairs signed rank test) were used to compare quantitative variables across two groups. The Kruskal-Wallis test with Dunn’s multiple comparison test was used to compare quantitative variables across three or more groups. A significance level of *p* < 0.05 was set for statistical associations (*p < 0.05; **p < 0.01; ***p < 0.001; ****p < 0.0001).

## Results

### Clinical Features of Six CVID Patients Affected by GLILD

We have selected 100 subjects from our cohort of 122 adults and pediatrics with CVID patients, for this study. Twenty-two patients were excluded as lung CT scans were not available. Among the included CVID patients, 6 out of 100 (6.0%) received a diagnosis of CVID-associated GLILD. All GLILD-affected patients were alive at the end of the present study. Table 1 summarizes demographical and clinical data of the GLILD-affected CVID patients. Age at CVID onset was variable, with 3/6 patients presenting CVID symptoms already in childhood; nonetheless, CVID diagnosis was performed only in one patient under the age of 16 years (median age at CVID onset 17.5 years, mean age at CVID onset 16.2 years, IQR 4.3–20.45 years; median age at CVID diagnosis 20.5 years, mean age at CVID diagnosis 23.55 years, IQR 11.3–27.5 years). Symptoms of CVID onset in the six GLILD-affected patients were variable: interestingly, 5 out of 6 patients presented non-infectious complications as first symptom of CVID such as autoimmune manifestations affecting the hematological and endocrine systems or chronic lymphoproliferation. Both autoimmunity and lymphoproliferation occurred in all patients before GLILD diagnosis. At GLILD diagnosis, 5/6 patients were already put on IgRT, while the remaining patient (P4) started IgRT at the time of GLILD diagnosis which was concurrent with CVID diagnosis. In all patients, GLILD diagnosis was established in adult age (median and mean age at GLILD diagnosis 36.5 and 34.9 years, respectively). Interestingly, only in a single patient (P4), GLILD diagnosis was shortly after the onset of CVID manifestations, while in all the other patients, GLILD diagnosis occurred at a median interval of 15.95 years after CVID onset (mean 18.7 years, IQR 0.4–24.75 years).

**Table 1:** Demographical and clinical data of the six GLILD-affected CVID patients

	P1	P2	P3	P4	P5	P6
Gender	M	F	M	F	F	M
Current age (years)	56.0	22.6	46.9	17.8	40.6	39.7
Age at CVID onset (years)	28.2	8.9	4.3	15.6	20.8	19.4
Age at CVID diagnosis (years)	30.0	11.3	43.0	16.0	21.0	20.0
Age at GLILD diagnosis (years)	55.5	21.3	44.5	16.0	35.6	36.5
Delay GLILD diagnosis—CVID onset (years)	27.3	12.4	40.2	0.4	14.8	17.1
Delay GLILD diagnosis—CVID diagnosis (years)	25.5	10	1.5	0	14.6	16.5
Family history for IEIs	–	–	–	–	–	–
Smoking habits	–	–	–	–	–	–
Work exposure	–	–	–	–	–	–
Manifestation of CVID onset	RRTI	AIHA	ITP	NH lymphoma	Optic neuritis	ITP
Respiratory infections	+	+	+	–	+	+
Other infections	Ocular toxoplasmosis	Chronic CMV	Chronic sinusitis	Recurrent HSV-1 stomatitis	–	–
Autoimmunity	+	+	+	–	+	+
AIHA	–	+	+		–	–
ITP	–	+	+		–	+
Enteropathy	+	+	+		–	+
Other	–	Follicular psoriasis	–		Hypothyroidism	Psoriasis
Hepato-splenomegaly	+	+	+	–	+	+
Chronic lymphadenopathies	+	+	+	–	+	+
Malignancy	–	–	–	+	–	–
Type of IgRT	fSCIG	IVIG	fSCIG	fSCIG	fSCIG	fSCIG
Dose and frequency of IgRT	400 mg/kg/28 days	400 mg/kg/28 days	400 mg/kg/28 days	400 mg/kg/28 days	400 mg/kg/28 days	200 mg/kg/15 days
Antimicrobial prophylaxis	Atovaquone	TMP-SMX, valganciclovir	–	TMP-SMX, aciclovir	–	–

*AIHA* autoimmune hemolytic anemia, *CMV* cytomegalovirus, *CVID* common variable immunodeficiency, *fSCIG* facilitated subcutaneous immunoglobulin, *GLILD* granulomatous lymphocytic interstitial lung disease, *HSV-1*, Herpes Simplex Virus 1, *IgRT* immunoglobulin replacement treatment, *ITP* immune thrombocytopenia, *IVIG* intravenous immunoglobulin, *NH* non-Hodgkin, *RRTI* recurrent respiratory tract infections, *TMP-SMX* trimethoprim-sulfamethoxazole

"+": present; "-": absent

Prior to establishing GLILD diagnosis, all patients reported chronic cough, and 4/6 suffered from excessive sputum production. Dyspnea during physical activity was reported by all patients, while 2 of them also presented dyspnea at rest (33.3%). GLILD diagnosis was based on clinical data, lung CT scans findings and PFT results. All patients displayed ground-glass opacities and parenchymal micronodules/nodules, while interlobular septal thickening, airspace consolidation, and reticular pattern were present in four of them. Other non-pulmonary features associated with GLILD, such as hilar or mediastinal lymphadenopathies and splenomegaly, were observed in five patients. Lastly, bronchiectasis was a manifestation observed in half of the patients. PFTs analysis showed an important reduction of TLC, DL_CO_, and K_CO_ in all patients, while FVC and FEV_1_ were normal or modestly altered. Two patients (P3, P5) underwent also bronchoalveolar lavage for microbiological analysis (which tested negative) and P5 underwent a lung biopsy, which revealed lymphocytic infiltrates with non-necrotizing granulomata. Data regarding GLILD diagnosis are summarized in Table 2.

**Table 2:** Clinical and diagnostic features that lead to GLILD diagnosis in six CVID patients

GLILD symptoms	
Chronic cough	6 (100%)
Excessive sputum	4 (66.6%)
Dyspnea on exertion	6 (100%)
Dyspnea at rest	2 (33.3%)
Loss of weight	2 (33.3%)
Lung CT features	
Airspace consolidation	4 (66.6%)
Bronchiectasis	3 (50.0%)
Ground-glass opacities	6 (100%)
Interlobular septal thickening	4 (66.6%)
Lymphadenopathies	5 (83.3%)
Mucus pluggings	1 (16.6%)
Nodules or micronodules	6 (100%)
Splenomegaly	5 (83.3%)
Reticular pattern	5 (83.3%)
PFTs	
FVC % of predicted <100%	4 (66.6%)
FEV_1_ % of predicted <100%	3 (50.0%)
TLC % of predicted <100%	6 (100%)
DL_CO_ % of predicted <100%	6 (100%)
K_CO_ % of predicted <100%	6 (100%)
Bronchoalveolar lavage^a^	
Microbiological cultures positive	0 (0%)
Lung biopsy^b^	
Lymphocytic infiltrates with non-necrotizing granulomata	1 (100%)

^a^2 patients performed bronchoalveolar lavage

^b^1 patient performed lung biopsy

*CT* computed tomography, *DL*_*CO*_ diffusion capacity of the lung for carbon monoxide, *FEV*_*1*_ forced expiratory volume in 1 s, *FVC* forced vital capacity, *GLILD* granulomatous lymphocytic interstitial lung disease, *K*_*CO*_ Krogh index, *PFTs* pulmonary function tests, *TLC* total lung capacity

Data from immunological evaluation performed at the time of GLILD diagnosis are shown in Table S1 in the Online Resource. All patients displayed reduced to absent serum IgA levels; raised serum IgM levels were detected in 1 out of 6 patients. T-cell count was below normal ranges in 2 out of 6 patients even though CD4^+^ lymphopenia was observed only in patient P2. Looking at detailed lymphocyte subsets analysis, a reduction on the naïve and RTE CD4^+^ T lymphocyte was observed in 4 out of 6 patients, whose memory cells (CD45RA^-^CCR7^+^ and CD45RA^-^CCR7^-^) represented the vast majority of the whole maternal subset (83.3–96.2%); in addition, in 3 out of 6 patients, a skewed maturation toward CD8^+^ terminally differentiated subsets was noted. On B-cell subsets, 5 out of 6 patients displayed low naïve cell counts (1.4–39.6 cells/μl), 4 of which associated with a very weak bone marrow output (0.3–7.2 cells/μl). The whole cohort presented low to absent CD19^+^ switched memory cells (0–0.4 cells/μl), seen as IgD^-^IgM^-^CD27^+^CD21^hi^ cells, while terminal differentiated cells were detectable only in patient P6. An expansion of CD19^hi^CD21^lo^ cells was reported in 4 out of 6 patients, which in 3 cases constituted by far the most consistent subset of the whole B-cell compartment. Genetic analysis by means of targeted NGS was negative in the entire cohort.

### First-Line Rituximab Monotherapy Regimen

All GLILD-affected patients received anti-CD20 monoclonal antibody (Rituximab) intravenous infusions as first-line treatment. None of the patients had received immunosuppressive or immunomodulating treatments in the 5 years before the beginning of Rituximab that was administered at 375 mg/m^2^ monthly for 6 infusions followed by an every 3-month maintenance scheme. At the end of the present study, 5 out of 6 patients are still under treatment with every 3-month Rituximab infusions. Follow-up is variable, ranging from 0.5 to 5.0 years (median 2.1 years, mean 2.4 years, IQR 1.4–3.0 years). None of the treated patients experienced severe or serious adverse events following Rituximab, infusions and no other immunosuppressive/immunomodulator agents were implemented to achieve GLILD remission. During Rituximab treatment, none of the GLILD-affected patients reported an increased rate of infectious episodes nor severe/invasive infections, and no additional antimicrobial treatments were needed.

### Rituximab Monotherapy Led to Complete GLILD Remission in the Whole Cohort of Patients

**Fig. 1:**
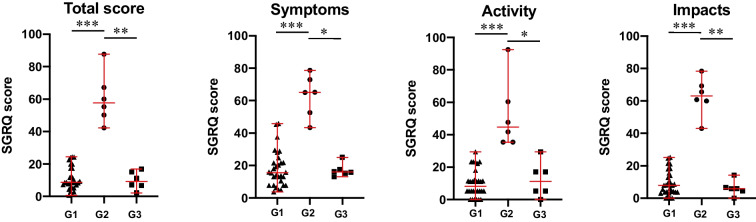
Symptomatic burden and quality of life by means of St. George’s Respiratory Questionnaire (SGRQ) results in common variable immunodeficiency (CVID) patients without GLILD diagnosis (G1, black triangles) and GLILD-affected CVID patients before Rituximab (RTX) treatment (G2, black circles) and after RTX treatment (G3, black squares). Red lines represent median and interquartile ranges (*p < 0.05; **p < 0.01; ***p < 0.001; ****p < 0.0001)

Symptom burden and QoL alteration were evaluated by means of the SGRQ. The SGRQ results were compared to the results obtained from 30 CVID patients without GLILD diagnosis, age- and sex-matched with the GLILD-affected CVID patients; in addition, no statistically significant differences regarding clinical manifestations (infectious episodes, autoimmunity manifestations, chronic lymphoproliferation, and malignancy) were noted between the two groups (see Table S2 in this article’s Online Resource). At baseline, CVID patients with GLILD diagnosis presented statistically significant higher results both in the SGRQ total score and in the three single items (symptoms, activity, impacts) (*p* = 0.0005, 0.0004, 0.0003, and 0.0005, respectively, Fig. 1). After 6 months from the first Rituximab infusion, all the treated GLILD-affected patients did not report any respiratory symptoms, and they were able to return to conduct their normal daily activities and attend to work or school. A mean rank difference of 22.25 points on the SGRQ total score was observed (*p =* 0.0050), as well as a reduction of all the three single components of the SGRQ score (symptoms, activity, impacts; *p* = 0.0176, 0.0165, and 0.0031, respectively, Fig. 1).

**Fig. 2:**
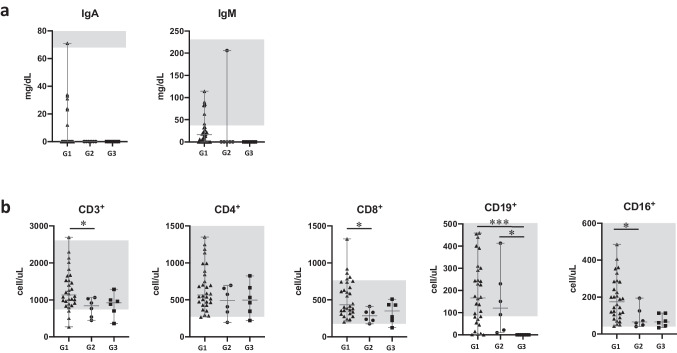
**a** Serum IgA and IgM levels and **b** main lymphocyte subsets comparison in CVID patients without GLILD diagnosis (G1, black triangles) and GLILD-affected CVID patients before Rituximab (RTX) treatment (G2, black circles) and after RTX treatment (G3, black squares). Red lines represent median and interquartile ranges. Gray boxes represent normal range for healthy subjects (*p < 0.05; **p < 0.01; ***p < 0.001; ****p < 0.0001)

At GLILD diagnosis, no differences were observed between the two groups for IgA and IgM serum levels (Fig. 2a). Looking at the main lymphocyte subsets, the GLILD-affected cohort presented a statistically significant reduction in terms of absolute numbers of CD3^+^, CD8^+^, and CD56^+^CD16^+^ lymphocytes (*p* = 0.0343, 0.0342, and 0.0184 respectively) (Fig. 2b). Despite the presence of some well-defined trends, a more detailed analysis of CD4^+^, CD8^+^, and CD19^+^ lymphocyte subpopulations did not highlight additional differences between the two cohorts of CVID patients (see Fig. S2 in this article’s Online Resource). We re-evaluated the immunological parameters of the GLILD patients after 6 months from the beginning of Rituximab infusions. No differences were observed in terms of serum immunoglobulin levels before and after the Rituximab treatment (Fig. 2a). As one may expect, after 6 months from the beginning of Rituximab treatment, we observed a statistically significant reduction of total CD19^+^ B cells (*p* = 0.0256) and B-cell subpopulations (Fig. 2b and Fig. S2 in this article’s Online Resource), and none of the patient displayed B-cell compartment reconstitution. No other remarkable differences were observed in the other lymphocyte subpopulations (Fig. S2 in this article Online Resource).

**Fig. 3:**
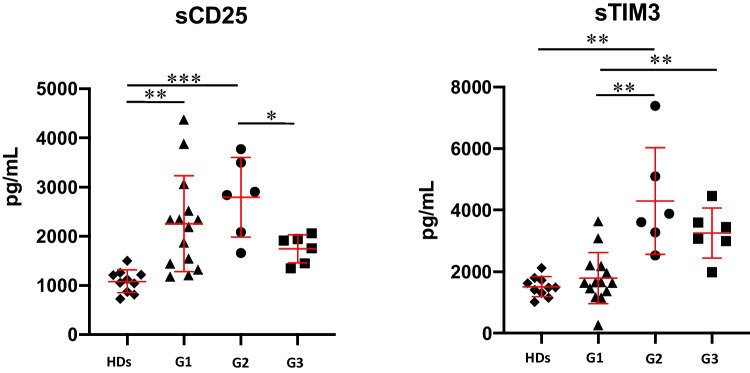
sCD25 and sTIM-3 serum levels in healthy donors (HDs—black diamonds), CVID patients without GLILD diagnosis (G1, black triangles) and GLILD-affected CVID patients before Rituximab (RTX) treatment (G2, black circles) and after RTX treatment (G3, black squares). Red lines represent median and interquartile ranges (*p < 0.05; **p < 0.01; ***p < 0.001; ****p < 0.0001)

T-cell activation and exhaustion serum markers were evaluated by means of serum concentration of sCD25 and sTIM-3 in HDs (*n* = 10), CVID patients without GLILD diagnosis (*n* = 14, randomly chosen from the 30 CVID patients control cohort), and the 6 GLILD-affected CVID patients (Fig. 3). Compared to HDs and CVID patients without GLILD diagnosis, GLILD-affected CVID patients displayed a statistically significant higher concentration of sTIM-3 (*p* = 0.0011 and 0.0086, respectively). Regarding sCD25, both CVID patients without GLILD diagnosis and CVID with GLILD diagnosis presented statistically significant higher concentration when compared to HDs (*p* = 0.0016 and 0.0004, respectively), but without differences between these two different cohorts of CVID patients. After 6 months from the start of treatment, a reduction of sCD25 serum concentration was observed (*p* = 0.0260); although not significant, sTIM-3 serum concentration presented a similar decreasing trend and reduced the strength of significance when compared to CVID patients without GLILD diagnosis (Fig. 3).

**Fig. 4:**
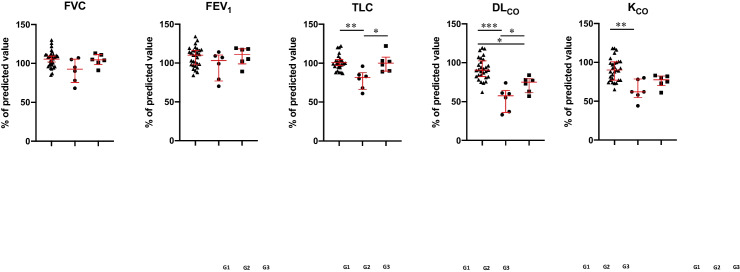
Pulmonary function tests (PFTs) expressed as percentage of predicted value in CVID patients without GLILD diagnosis (G1, black triangles) and GLILD-affected CVID patients before Rituximab (RTX) treatment (G2, black circles) and after RTX treatment (G3, black squares). Red lines represent median and interquartile ranges (*p < 0.05; **p < 0.01; ***p < 0.001; ****p < 0.0001)

Looking at PFTs at baseline, we observed a statistically significant reduction in TLC (*p* = 0.0020), DL_CO_ (*p* = 0.0002), and K_CO_ (*p* = 0.0035) when compared to the control cohort of CVID patients without GLILD diagnosis (Fig. 4). After 6 months from the beginning of Rituximab administration, a statistically significant increase in TLC (*p* = 0.0221) and DL_CO_ (*p* = 0.0411) was observed (Fig. 4). Although not significative, a similar trend has been noted also in FVC, FEV_1_, and K_CO_ when compared to CVID patients without GLILD diagnosis.

**Fig. 5:**
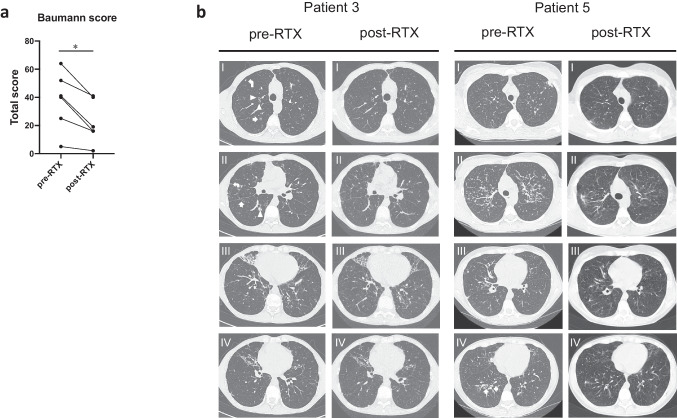
**a** Baumann score comparison in the 6 GLILD-affected CVID patients before (pre-RTX) and after (post-RTX) Rituximab treatment (*p < 0.05; **p < 0.01; ***p < 0.001; ****p < 0.0001). **b** Exemplificative lung CT scans from P3 and P5 before and after 6 months of Rituximab treatment. P3 before treatment (pre-RTX, first column): (I) In the right upper lobe, bronchial wall thickening (arrowheads) of the segmental branches is observed; focal area of ground-glass opacity (arrow) and a small parenchymal nodule in the anterior segment (curve arrow) can be detected; (II) parenchymal consolidations (arrowhead), peripheral ground-glass opacity (curve arrow), and linear scars (arrow) can be depicted; (III–IV) cylindrical bronchiectasis in the middle lobe and lower lingular segment can be observed. P3 after treatment (post-RTX, second column): (I–IV) bronchial wall thickening, the focal area of ground-glass opacity, and the small parenchymal nodule are no longer evident. Unchanged bronchiectasis in the middle lobe and in the lingula. P5 before treatment (pre-RTX, third column): (I) area of parenchymal consolidation in the left lung, at the periphery, (II) numerous small nodules in both lungs can be observed, and (III–IV) bronchial wall thickening in the segmental branches of the inferior right lobe (arrows). P5 after treatment (post-RTX, fourth column): (I–IV) Reduction in the number of nodules located in both lungs in these sites, sporadic areas of alveolar consolidation can be detected

For each patient, CT scans were scored with the Baumann method at GLILD diagnosis and after 6 months of Rituximab treatment. At diagnosis, the mean score was 37.83 (median 40.50); after Rituximab treatment, we observed a statistically significant reduction of the score for each single patient, with a mean score of 23.33 (median 17.50) (*p* = 0.0312) (Fig. 5a). Exemplificative lung CT scans from P3 and P5 before and after 6 months of Rituximab treatment are pictured in Fig. 5b.

## Discussion

Here, we report on the results of a single-center retrospective study investigating the efficacy of Rituximab in treating CVID-associated GLILD. Based on clinical and radiological data from lung CT scans, a diagnosis of GLILD was achieved in 6.0% of our CVID cohort. GLILD prevalence is reported to vary between 8 and 20% depending on the study setting, and our data is in line with the literature [4]; however, we could possibly have underestimated the actual prevalence of GLILD in our cohort of CVID patients as 22 of them have been excluded from this study because lung CT scans data were not available. Common radiological features that led to GLILD diagnosis were ground-glass opacities and nodules/micronodules, which were present in all of our GLILD-affected patients; additional recurrent features such as airspace consolidation, interlobular septal thickening, hilar/mediastinal lymphadenopathies, and splenomegaly were also present in two-thirds of the patients. Taken together, these radiological findings have already shown to be highly specific for GLILD diagnosis, in particular when comprehensively evaluated using a scoring system such as the Baumann score [14]. Considering that lung biopsy carries an increased risk of morbidity and mortality in interstitial lung disease patients [15], in selected situations such as tertiary referral centers where experienced physicians deal with IEI-affected patients, invasive procedures could be overcome when clinical, radiological, and PFTs data are strongly suggesting the presence of GLILD.

Many patients in our cohort presented CVID-related symptoms since pediatric age, but a diagnosis of CVID was done in the vast majority of patients in adult age. This might have played a role in exposing patients to persistent microbiological stimuli that together with impaired antigen clearance may at least partially contribute to the development of immune dysregulatory manifestations [16]. Prior to GLILD diagnosis, all 6 CVID patients had already developed a heterogenous and complex spectrum of complications, in particular autoimmune phenomena and chronic lymphoproliferation. Hypersplenism and autoimmune manifestations have already been reported as strong risk factors for GLILD diagnosis in CVID patients [17, 18], and this must be kept in mind when evaluating CVID patients with complex clinical history, as CVID patients with non-infectious complications seem to be more prone to develop interstitial lung disease and therefore must be screened accordingly.

Increased IgM serum levels have been reported in GLILD-affected CVID patients, and an expansion of IgM memory B cells has been proposed as part of the pathogenetic mechanism beyond granulomata and lymphocytic infiltrates in the lung [19]; in contrast with these findings, only one of our patients displayed raised IgM serum levels. We also observed an expansion of CD19^hi^CD21^lo^ B cells in 66.6% of GLILD-affected patients, although the reduced size of our cohort could not lead to statistical significance. Nevertheless, CD19^hi^CD21^lo^ expansion has already been reported in GLILD-affected CVID patients and therefore should be highly considered as a potentially warning sign for GLILD development risk [20].

A recent publication by Fraz and colleagues analyzed various biomarkers in GLILD patients [21]: we confirmed increased sTIM-3 levels when compared to healthy donors and CVID patients without GLILD diagnosis. Conversely, in our cohort, sCD25 serum levels, a marker of T-cell activation, did not differ between CVID patients without or with GLILD diagnosis, but this might be due to the reduced size of our cohort.

In contrast with other published cohorts of GLILD patients [7], genetic analysis in our cohort did not reveal monogenic defects linked to immune dysregulation in CVID.

All our patients were treated with Rituximab monotherapy as first-line treatment, without the needs of systemic steroids nor additional chemotherapy. Anti-CD20 monoclonal antibody was administered at 375 mg/m^2^/months for 6 months (induction) and later continued at the same dosage every 3 months (maintenance). Excluding a case report already published by our group [22], Rituximab monotherapy for GLILD has been reported in a total number of 19 patients [6, 23–27]. Comparing the therapeutical scheme, all the other reported patients have been treated with Rituximab 375 mg/m^2^ weekly infusions for 4 weeks, with a 4- to 6-month interval between each cycle. While a comparison between the different therapeutical schemes should require a different study design, it is interesting to observe that the patient reported by Zdziarsky and colleagues that was initially treated with low dosage Rituximab monotherapy (150 mg/m2 weekly for 6 weeks) presented GLILD relapse 6 months after the end of the treatment but achieved complete remission after increasing the dosage to the standard dose of 375 mg/m^2^ [23]. We therefore strongly suggest that Rituximab-based treatment for GLILD should follow the standard dosage of 375 mg/m2, despite the chosen interval of administration.

Treatment efficacy was evaluated comparing SGRQ score results, immunological data, PFT results, and lung CT scan findings (using the Baumann score) at GLILD diagnosis and after 6 months of treatment. The use of the SGRQ has enabled us to quantify the impact of GLILD in terms of symptoms, impairment of daily activities, and quality of life. When used for assessing treatment response in asthma or chronic obstructive pulmonary disease, a mean change score of 12 units is associated with very efficacious treatment [9]. After 6 months from the beginning of Rituximab treatment, we observed a mean change of 50.6 units (+ 5.4); since we have also reported an important reduction of the Baumann score and an amelioration of the PFTs parameters, such a large decrease of the SGRQ score might be explained on one hand by a reversion of the lung parenchyma alterations (e.g., granulomata, lymphocytic infiltrates, ground-glass opacities) that does not occur in other types of chronic lung disease where treatment is not targeting the potential pathogenic mechanisms and thus is not capable of reverting the damages.

Lymphocyte subset analysis after Rituximab treatment showed only minor modifications. As expected when using B-cell depleting therapies, we observed absence of peripheral B cells in all treated patients, while no other significant differences on the remaining lymphocyte subpopulations were noted. On the contrary, serum sCD25 and sTIM-3 levels showed a decline upon Rituximab treatment, suggesting a possible indirect effect of anti-CD20 monoclonal antibodies on T-cell activation and exhaustion. To the best of our knowledge, this is the first time that sCD25 and sTIM-3 are evaluated both at GLILD diagnosis and after treatment. Although available only in research settings, sCD25 and sTIM-3 could be used as GLILD biomarkers not only as an aid in GLILD diagnosis but also in disease activity monitoring during follow-up. In addition, if reproduced in other Rituximab-treated GLILD patients, these results could become fundamental when deciding the therapeutical regimen, as additional chemotherapy or immunomodulators targeting T cells might not be needed: in the Verbsky’s study [7], combined immunosuppressive therapy with Rituximab and antimetabolite (i.e., azathioprine or mycophenolate mofetil) resulted in long-lived remission in the vast majority of the treated patients; however, leukopenia o lymphopenia, increased rate of infectious episodes, and septicemia occurred, suggesting that combined therapy regimen might cause (at least in some patients) excessive immunosuppression.

In our cohort, PFTs have shown an important role both in highlighting a restrictive pattern, which is typical of GLILD, and in monitoring disease remission after Rituximab treatment. PFTs are a reproducible and noninvasive procedure already recommended but not always performed for CVID patients’ follow-up [28]. Therefore, our results underline the importance of monitoring lung function in CVID patients, as early identification of CVID patients presenting a decline in lung function warrants further investigations such as lung CT scans. Furthermore, the Baumann score has already been used for evaluating the extent of GLILD with good reproducibility among trained radiologists [12]. A decrease of the Baumann score followed the same trend of clinical and PFTs improvement; besides its valuable application in research settings, our data suggest that the Baumann score may be applied in clinical practice as a tool for screening CVID patients for GLILD and monitoring GLILD activity.

Treatment approach for GLILD is variable and mainly based on the single-center own experience. Systemic corticosteroids are usually proposed as first-line treatment; the large cohort recently reported by Smits and colleagues [29] revealed that high-dose corticosteroids are needed to achieve remission and patients with relapse tend to show a worse disease response when retreated with corticosteroids. In addition, opportunistic infections and steroid-related skeletal complications were reported [29]. On the other hand, as reported by Verbsky [7], combined immunosuppressive treatment induced radiological remission in the majority of patients even though GLILD relapse occurred when immunosuppressive treatment was stopped, and patients might present increased infectious episodes caused by targeting both B and T cell. On the contrary, Rituximab monotherapy seems safer as no increased rate of infectious episodes was observed in our cohort as well as no drug-related adverse events, with all the treated patients experiencing complete disease remission after 6 months of treatment.

This study presents some limitations. First of all, the retrospective nature of our study and the use of a tertiary academic single center represent another obvious bias. In addition, anti-CD20 monoclonal antibody was the only prescribed treatment in our cohort, and therefore, we could not compare the outcomes with other therapeutical options; likewise, in our cohort, only adult GLILD-affected CVID patients have been studied and treated, and therefore, our results may not be replicated with pediatrics patients. Patients were evaluated for treatment response after 6 months of treatment, but no additional related evaluations were performed after this interval—all patients remained in stable conditions. Finally, increasing the size of the cohort of patients could lead to more robust results.

In conclusion, first-line monotherapy with Rituximab displayed high efficacy in disease remission in the entire treated cohort, with improvement of symptoms and amelioration of quality of life, as well as restoration of PFTs and resolution of lung CT scan findings. Both preclinical studies on GLILD pathogenesis and multicenter randomized clinical trials to ultimately assess the efficacy of Rituximab therapy over steroids or other immunosuppressive agents are warranted to further understand this rare yet fatal complication of CVID and develop strong evidence-based therapeutical guidelines for affected patients.

### Supplementary Information

ESM 1 (PDF 560 kb)

## Data Availability

The datasets generated and/or analyzed during the current study are available from the corresponding author on reasonable request.

## Abbreviations

CVID: Common variable immunodeficiency
GLILD: Granulomatous lymphocytic interstitial lung disease
QoL: Quality of life
UK-PID: United Kingdom Primary Immunodeficiency
PFTs: Pulmonary function tests
CT: Computed tomography
IgRT: Immunoglobulin replacement treatment
SGRQ: St. George’s Respiratory Questionnaire
RTE: Recent thymic emigrants
NGS: Next-generation sequencing
sCD25: Soluble IL-2Rα chain/CD25
sTIM-3: Soluble T-cell Ig and mucin domain-containing protein 3
ERS: European Respiratory Society
FVC: Forced vital capacity
FEV_1_: Forced expiratory volume in 1 s
TLC: Total lung capacity
DL_CO_: Diffusion capacity of the lung for carbon monoxide
AV: Alveolar volume
K_CO_: Krogh index
PACS: Picture archiving and communication system
