# Supplementary material for: Rituximab Monotherapy Is Effective as First-Line Treatment for Granulomatous Lymphocytic Interstitial Lung Disease (GLILD) in CVID Patients
Source: J Clin Immunol. 2023 Sep 27;43(8):2091–103. doi: 10.1007/s10875-023-01587-4 (PMC10661825; doi:10.1007/s10875-023-01587-4)

Supplemental Table S1. Laboratory and immunological data of the six GLILD-affected CVID patients

	**P1**	**P2**	**P3**	**P4**	**P5**	**P6**
Red Blood Cells ($x10^6$/uL) (4.50 - 5.50)	5.1	4.0↓	6.2↑	4.1↓	5.4	4.9
Hemoglobin (g/dL) (14.0 - 18.0)	14.8	8.5↓	14.8	13.8↓	10.5↓	14.2
Platelets ($x10^3$/uL) (130 - 400)	145	101↓	112↓	144	197	169
White Blood Cells ($x10^3$/uL) (4.00 - 10.80)	5.3	3.38↓	4.92	4.5	7.6	7.3
Lymphocytes ($x10^3$/uL) (0.90 - 4.00)	1.59	0.57↓	0.81↓	0.79↓	1.04	1.56
Neutrophils ($x10^3$/uL) (1.50 - 8.00)	2.97	2.53	3.75	2.91	5.77	4.97
IgA (mg/dL) (70 - 400)	<10↓	<10↓	<10↓	<10↓	<10↓	<10↓
IgM (mg/dL) (40 - 230)	<4↓	<4↓	<4↓	<4↓	<4↓	206
T cells ($CD3^+$) (cell/uL) (721 - 2562)	1034	445↓	765	538↓	915	1064
T cells ($CD3^+$) (%) (57.1 - 87.6)	65.7	82.3	79.1	77.8	88.0↑	71.4
$CD3^+CD4^+$ (cell/uL) (273 - 1882)	574	195↓	407	337	655	695
$CD3^+CD4^+$ (%) (28.5 - 65.6)	36.5	36.1	42.1	48.8	63.5	46.6
HLA-DR^+ (%) (1.6 - 12.2)	3.0	13.3↑	1.7	3.3	6.3	11.8
Naive ($CD45RA^+CCR7^+$) (%) (20.4 - 63.6)	10.1↓	2.7↓	13.5↓	71.6↑	42.7	5.8↓
RTE ($CD45RA^+CCR7^+CD31^+$) (%) (11.4 - 48.1)	5.8↓	2.1↓	9.2↓	56.1↑	30.9	4.1↓
Central memory ($CD45RA^-CCR7^+$) (%) (18.7 - 46.2)	73.4↑	19.6	39.9	12.9↓	33.3	45.0
Effector memory ($CD45RA^-CCR7^-$) (%) (7.1 - 38.0)	15.8	76.6↑	43.3↑	10.4	22.4	48.4↑
Terminally differentiated ($CD45RA^+CCR7^-$) (%) (0.3 - 9.1)	0.7	1.1	3.2	5.1	1.6	0.8
$CD3^+CD8^+$ (cell/uL) (177 - 783)	410	237	331	176	223	334
$CD3^+CD8^+$ (%) (10.5 - 37.7)	26.1	43.8↑	34.2	25.4	21.5	22.4
HLA-DR+ (%) (2.7 - 31.7)	6.5	31.5	2.0↓	10.2	18.1	29.5
Naive ($CD45RA^+CCR7^+$) (%) (13.1 - 66.5)	13.9	1.4↓	10.6↓	49.2	38.4	16.2
Central memory ($CD45RA^-CCR7^+$) (%) (2.6 - 24.5)	7.4	0.4↓	1.4↓	0.5↓	2.9	5.5
Effector memory ($CD45RA^-CCR7^-$) (%) (10.1 - 47.4)	10.6	32.4	22.9	19.5	18.4	42.1
Terminally differentiated ($CD45RA^+CCR7^-$) (%) (5.2 - 63.5)	68.1↑	65.8↑	65.1↑	30.8	40.2	36.1
$CD4^+/CD8^+$	1.4	0.82	1.23	1.92	2.9	2.08
$CD4^-CD8^-$ TCR γ/δ^+ (%) (0.9 - 11.2)	6.9	4.3	4.7	2.8	2.7	0.5↓
B cells ($CD19^+$) (cell/uL) (86 - 648)	413	22↓	151	91	10↓	230
B cells ($CD19^+$) (%) (5.8 - 22.1)	26.3↑	4.2↓	15.6	13.1	1.0↓	15.4
RBE ($CD38^{hi}CD10^+$) (%) (2.1 - 26.1)	1.5↓	32.7↑	0.3↓	69.3↑	3.1	7.7
Naive ($IgD^+IgM^+CD27^-CD21^{hi}$) (%) (33.7 - 74.0)	9.6↓	64.5	22.0↓	27.6↓	14.4↓	52.8
$CD19^{hi}CD21^{lo}$ (%) (1.4 - 13.6)	78.1↑	3.1	65.7↑	1.5	61.6↑	33.8↑
Switched memory ($IgD^-IgM^-CD27^+CD21^{hi}$) (%) (2.8 - 23.4)	0.1↓	0.0↓	0.0↓	0.0↓	1.7↓	0.1↓
IgM memory ($IgD^+IgM^+CD27^+CD21^{hi}$) (%) (5.1 - 25.5)	11.8	0.0↓	11.5	0.0↓	19.4	2.9↓
Terminally differentiated ($CD38^{hi}CD27^+CD20^-$) (%) (0.2 - 8.1)	0.0↓	0.0↓	0.0↓	0.0↓	0.0↓	0.1↓
NK cells ($CD3^-CD16^+CD56^+$) (cell/uL) (40 - 741)	125	71	49	60	40	194
NK cells ($CD3^-CD16^+CD56^+$) (%) (3.4 - 28.4)	8.0	13.1	5.1	8.7	4.6	13

Supplemental Table S2. Demographical and clinical data of 30 CVID patients without GLILD diagnosis and 6 GLILD-affected CVID patients

	CVID patients without GLILD diagnosis (n=30)	CVID patients with GLILD diagnosis (n=6)	significance
Female, n (%)	18 (60.0%)	3 (50.0%)	*p* 0.6774
Age in years, mean (95% CI)	37.02 (32.90 – 41.15)	34.90 (19.61 – 50.19)	*p* 0.6858
Infection, n (%)	28 (93.3%)	6 (100.0%)	*p* >0.999
Autoimmunity, n (%)	14 (46.7%)	5 (83.3%)	*p* 0.1821
Lymphoproliferation, n (%)	21 (70.0%)	5 (83.3%)	*p* 0.6546
Malignancy, n (%)	3 (10.0%)	1 (16.7%)	*p* 0.5348

Supplemental Figure legend:

Fig. S1. Rituximab treatment protocol for GLILD affected patients, with baseline and treatment response evaluation. SGRQ, St. George's Respiratory Questionnaire; sTIM-3, soluble T cell Ig and mucin domain-containing protein 3; sCD25, soluble IL-2Rα chain/CD25; PFTs, pulmonary function tests; CT, computed tomography.

Fig. S2. (a) $CD4^+$, (b) $CD8^+$, and (c) $CD19^+$ lymphocyte subpopulations comparison in CVID patients without GLILD diagnosis (G1, black triangles) GLILD affected CVID patients before Rituximab (pre-RTX) treatment (G2, black circles) and after Rituximab (post-RTX) treatment (G3, black squares). Red lines represent median and interquartile ranges. Grey boxes represent normal range for healthy subjects. (* $p < 0.05$; ** $p < 0.01$; *** $p < 0.001$; **** $p < 0.0001$).

Supplemental Fig. S1

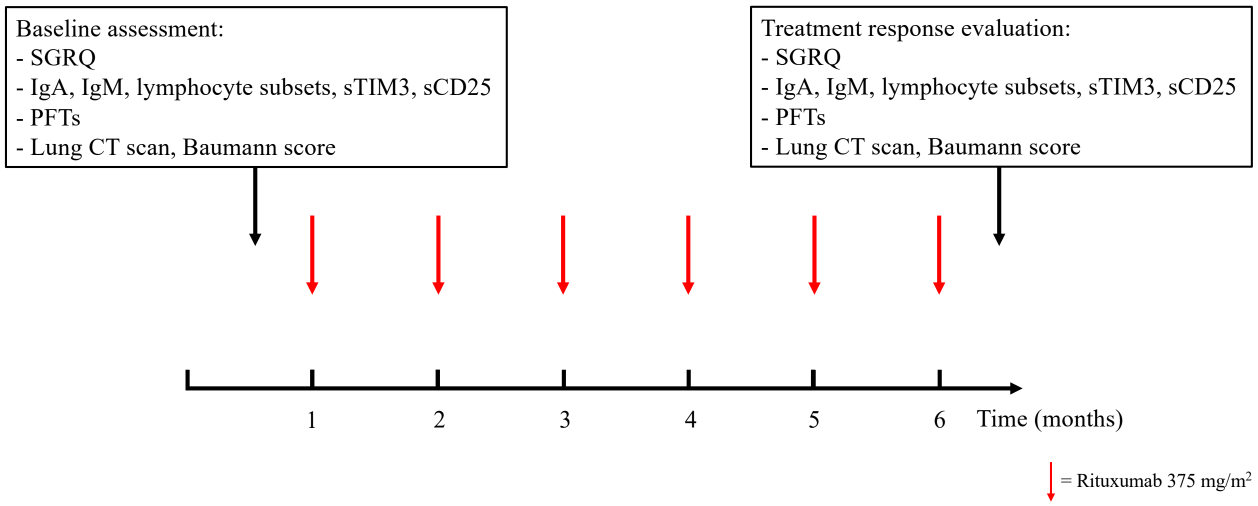

Supplemental Fig. S2

Fig. S1

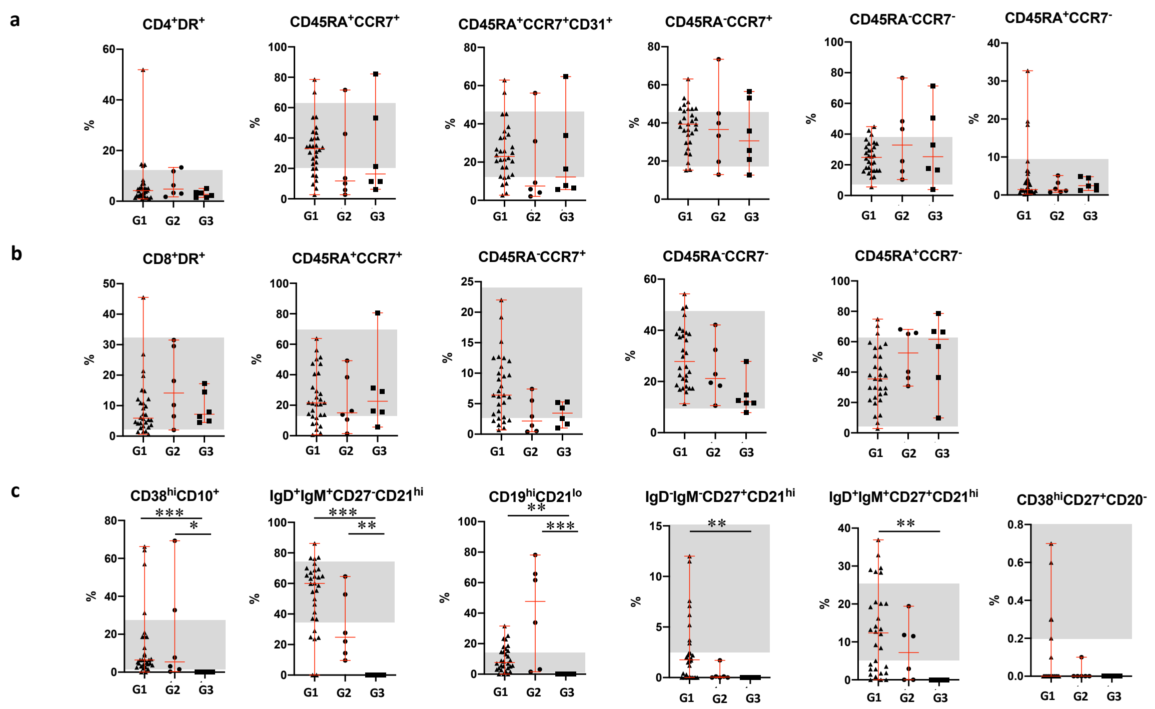

Supplement: Supplementary file 1 — (PDF 560 kb) [file 10875_2023_1587_MOESM1_ESM.pdf]
